# Tracers progress for positron emission tomography imaging of glial-related disease

**DOI:** 10.7555/JBR.36.20220017

**Published:** 2022-06-28

**Authors:** Haoran Jia, Tianwu Xie

**Affiliations:** Institute of Radiation Medicine, Fudan University, Shanghai 200032, China

**Keywords:** glia, positron emission tomography tracer, glioma, Alzheimer's disease, neuroinflammation

## Abstract

Glial cells play an essential part in the neuron system. They can not only serve as structural blocks in the human brain but also participate in many biological processes. Extensive studies have shown that astrocytes and microglia play an important role in neurodegenerative diseases, such as Alzheimer's disease, Parkinson's disease, Huntington's disease, as well as glioma, epilepsy, ischemic stroke, and infections. Positron emission tomography is a functional imaging technique providing molecular-level information before anatomic changes are visible and has been widely used in many above-mentioned diseases. In this review, we focus on the positron emission tomography tracers used in pathologies related to glial cells, such as glioma, Alzheimer's disease, and neuroinflammation.

## Introduction

The brain serves as the center of the nervous system composed primarily of two broad classes of cells: neurons and glial cells. Neurons send signals to specific target cells over long distances through axons. Most of the space in the brain is taken up by axons, which are often bundled together as nerve fiber tracts. Some axons are wrapped in a fatty insulating sheath of white myelin, making parts of the brain filled exclusively with nerve fibers appear as light-colored white matter, in contrast to the darker-colored grey matter that marks areas with high densities of neuron cell bodies. Although neurons are usually considered as the most important cells in the brain, glial cells, which are abundant in white matter, also play an essential part in the central nerve system (CNS).

Glial cells, also called glia or neuroglia, were originally believed to just provide structural support, and had a glia-neuron ratio (GNR) of 10:1, which are both proven wrong nowadays. It's now believed that GNRs vary in different regions of the brain and have an average number of less than 1:1^[1]^. Therefore, with more diversities than neurons, glial cells take part in 33% to 66% of the total brain mass^[2]^. In the CNS, glial cells consist of oligodendrocytes, astrocytes, ependymal cells, and microglia. They have four main functions: (1) to surround neurons and hold them in place; (2) to supply nutrients and oxygen to neurons; (3) to insulate one neuron from another; (4) to destroy pathogens and remove damaged or degenerative neurons.

### Glial cells and related diseases

Because of the heterogeneity of glial cells population, there are wide ranges of different neuro-pathologies and non-neural diseases related to glial disorders. Astrocytes and microglia play an important role in neurodegenerative disease, such as Alzheimer's disease (AD), Parkinson's disease (PD) and Huntington disease, as well as glioma, epilepsy, ischemic stroke and many kinds of infections^[3–5]^.

Neuroinflammation is an inflammatory response in the CNS, typically caused by cell damaging processes such as infection, traumatic brain injury, toxic metabolites and many other cues. During neuroinflammation, CNS immune glial cells will be activated and produce factors that mediate neuroinflammation process. There are both positive and negative aspects of neuroinflammation, depending on its intensity and duration^[6]^.

In cellular level, the immune activation in the brain is related to microglia and astrocytes, which generally aimed at evoking CNS immunity of removing harmful agents, repairing damaged tissue and maintaining homeostasis in non-pathological conditions. Under diseased conditions, both microglia and astrocytes would present phenotype changes from resting morphology to reactive phenotype, with function alternation and affecting the inflammatory process^[7–8]^. Chronic or exaggerated microglial activation may cause damage in the CNS and takes an important role in the process of synaptic dysfunction, neurogenesis inhibition, and neurodegenerative diseases^[9]^, such as AD, PD, amyotrophic lateral sclerosis, and frontotemporal dementia. In those diseases, the immune system may deactivate and even worse, neuroinflammation may contribute to disease processes. Inflammatory and neoplastic brain disorders also relate to neuroinflammatory reactions.

Glioma is another type of disease related to glial cells and is one of the most popular primary intracranial tumors which constitutes about 60% of all cerebral tumors^[10]^. According to the World Health Organization (WHO) classification of central nervous system tumors, gliomas can be divided into astrocytomas, oligodendrogliomas, and glioblastomas^[11]^. Although the cell origin of gliomas remains elusive, it may be closely related to all kinds of glial cells and glial progenitor cells^[12]^.

### Positron emission tomography imaging

Positron emission tomography (PET) is a functional imaging technique capable of detecting biochemical processes as well as the expression of some proteins. It provides molecular-level information before anatomic changes are visible, while computed tomography (CT) and magnetic resonance imaging (MRI) scans can only show images in the level of organs and tissues. PET imaging depends on radiolabelled molecular probes with different rates of uptake in various tissues. Therefore, selection of PET tracers is one of the key points for obtaining precise and meaningful diagnostic message. With PET imaging, glial phenotype can be distinguished *in vivo*. In this article, we summarize the tracers targeted to glial cells for PET imaging.

## Positron emission tomography tracers for neuroinflammation: selective to glial cells

Neuroinflammation is highly related to glial cells of inactive microglia and astrocytes with different phenotypes, where most of the imaging targets for neuroinflammation are located.

### Translocator protein

The first and most used neuroinflammation biomarker is translocator protein 18 kDa (TSPO, historically known as peripheral-type benzodiazepine receptor, PBR). TSPO is responsible for the translocation of cholesterol^[13]^, and involved in many physiological functions such as immunomodulation, cell respiration, and protein import^[14–17]^. TSPO is widely distributed throughout the body under physiological conditions. Its expression in the CNS, mainly in glial cells, is upregulated during neuroinflammation^[18]^, which makes it a reliable biomarker to reveal the activation of glial cells^[19]^.

#### The first generation

^**11**^**C-PK11195:** ^11^C-PK11195 was firstly developed as a TSPO tracer in 1984^[20]^ based on the increased binding of Ro5-4864 and PK11195 in microglial activation. PET using ^11^C-PK11195 has provided many information on glial activation in many different neurologic disorders, such as AD and PD^[21–22]^. A recent study shows multiple sclerosis patients and healthy controls have the same ^11^C-PK11195 plasma metabolization rate^[23]^. Although it can provide many information about microglia activation, its low signal-to-background ratio and high intra-subject variability cause a low sensitivity, thus hampering its clinical utility^[24–25]^. Based on this, to improve specific binding to TSPO and sensitivity, many second-generation tracers were developed.

#### The second generation

^**11**^**C-DPA-713:** ^11^C-DPA-713 (a pyrazolopyrimidine ligand) is one of the most promising second- generation TSPO traces with low lipophilicity and good affinity for TSPO^[26]^. It displays a higher signal-to-noise ratio than ^11^C-PK11195 with lower unspecific binding* in vivo*^[27]^. Its larger brain signal is proved in human studies^[28]^. Recent studies have shown that ^11^C-DPA-713 kinetic modeling possesses properties suitable for TSPO quantification with PET imaging^[29–30]^. Other pyrazolopyrimidine tracers developed from ^11^C-DPA-713, such as ^11^C-DPA-714^[31–32]^, VUIIS1008^[33]^ and compound 7^[34]^ are also under studies.

^**11**^**C-PBR28:** Developed from ^11^C-DAA1097 and ^11^C-DAA1106^[35]^,^ 11^C-PBR28 is an aryloxyanilide ligand with high affinity for TSPO^[36]^. Similar to ^11^C-DPA-713, ^11^C-PBR28 has a higher specific binding in human than ^11^C-PK11195^[37]^. A test in AD patients showed that ^11^C-PBR28 level changes with disease progressing and may be useful in longitudinal studies of AD^[38]^. It can also reveal the extensive glial activation in patients with chronic MCA stroke^[39]^ and semantic dementia^[40]^.

The second-generation TSPO tracer has a common limitation that hampers their use in clinical application— the sensitivity to a common polymorphism (rs6971), which means that subjects with same TSPO density may produce different PET signals^[41]^. Therefore, here goes the third generation TSPO PET tracers that may solve this difficulty and keep patients free from a pre-genotyping of TSPO.

#### The third generation

^**11**^**C-ER176:** ^11^C-ER176 is an isoquinoline analog ligand with a similar structure to ^11^C-PK11195^[42]^, which shows an adequate sensitivity to robustly image all three affinity genotypes. Although it still gives a lower signal in low-affinity gene type patients compared to high-affinity and missed-affinity patients, it’s high enough to be distinguished from non-specific tissue uptake^[43]^. ^11^C-ER176 also produces no radiometabolites that can pass through blood-brain-barrier (BBB)^[44]^. It has been successfully used to evaluate microglial activation in relation to neurodegenerative progression^[45]^.

^**18**^**F-GE180:** ^18^F-GE180 (also known as ^18^F-flutriciclamide) is another third-generation tracer with low sensitivity to rs6971^[46–47]^. It also shows a better signal-to-noise ratio and lower nonspecific binding. However, a recent study has shown that the advantages may be illusory due to its poor image quality and lack of ability to cross the BBB^[48]^.

### Monoamine oxidase B

^11^C-L-deprenyl: ^11^C-L-deprenyl is the most wildly studied non-TSPO radioligand targeting to monoamine oxidase B (MAO-B)^[49]^. The PET radioligand version of l-deprenyl or selegiline performs as a selective irreversible MAO-B inhibitor and binds with MAO-B enzyme in the temporal and the white matter in AD patients^[50]^, which indicates its ability to serve as a reliable PET tracer for imaging neuroinflammation .

### Summary

In the aspect of glial cell imaging, the specificity of TSPO PET for revealing microglial activation has been fully established. From the first generation TSPO PET tracer to the latest third generation, a higher sensitivity and lower off-target binding has been achieved, together with an adequately high binding for all rs6971 genotypes. Alternative strategies for the synthesis of the third-generation tracers have been studied^[45,51]^. Many other novel tracers based on them are also being tested^[52]^.

In neuroinflammation response, both TSPO and MAO-B are just one aspect that may reflect the immune progress. There are many other biomarkers that can be used to study neuroinflammation, such as glycogen synthase kinase 3 (GSK-3), reactive oxygen species (ROS), imidazoline-2 binding sites (I2BS), cyclooxygenase (COX), sphingosine-1-phosphate receptor 1 (S1P1), cannabinoid receptor 2 (CB2R), C-X3-C motif chemokine receptor 1 (CX3CR1), P2X ligand-gated ion channel type 7 (P2X7R), purinergic metabotropic 12 receptor (P2Y12R), receptor for advanced glycation endproducts (RAGE), Mer proto-oncogene tyrosine kinase (MerTK), and triggering receptor expressed on myeloid cells-1 (TREM1)^[18,53]^. For example, ^11^C-JNJ-54173717 is a promising tracer targeting to P2X7R which has been used to study amyotrophic lateral sclerosis^[54]^. Some of those tracers show more promising results for measuring neuroinflammation than TSPO-targeted tracers, although most of those tracers haven't been able to be studied in clinical trials. Some of the new targets have shown the selectivity between microglia and astrocytes. For instance, MAO-B and I2BS are mainly expressed by astrocytes, P2X7R, ROS, COX, S1P1, P2Y12R and TREM1 are mainly expressed by microglia^[53]^. PET tracers that selective to glial cells are summarized in ***Table 1***^[20–30,36–40,42–44,46–50,54]^.

**Table 1:** A summary for positron emission tomography tracers that selective to glial cells

Mainly expressed cells	Biomarkers	Tracers
Microglia and astrocytes	TSPO	^11^C-PK11195^[20–25]^, ^11^C-DPA-713^[26–30]^, ^11^C-PBR28^[36–40]^, ^11^C-ER176^[42–44]^, ^18^F-GE180^[46–48]^
	GSK-3	^11^C-SB-216763
	CB2R	^18^F-MA3
	MerTK	^18^F-HU16907
Astrocytes	MAO-B	^11^C-L-deprenyl^[49–50]^
	I_2_BS	^11^C-BU99008
Microglia	P2X_7_R	JNJ-54173717^[54]^
	ROS	^11^C-ascorbicacid
	COX	^11^C-PS13
	S1P1	^18^F-TZ4881
	P2Y_12_R	^11^C-2
	TREM1	^64^Cu-TREM1-mAb
^18^F: ^18^fluorine; ^11^C: ^11^carbon; ^64^Cu: ^64^cuprum; TSPO: translocator protein; GSK-3: glycogen synthase kinase 3; CB2R: cannabinoid receptor 2; MerTK: Mer tyrosine kinase; MAO-B: monoamine oxidase B; I_2_BS: imidazoline-2 binding sites; P2X_7_R: P2X ligand-gated ion channel type 7; ROS: reactive oxygen species; COX: cyclooxygenase; S1P1: sphingosine-1-phosphate receptor 1; P2Y_12_R: purinergic metabotropic 12 receptor; TREM1: triggering receptor expressed on myeloid cells-1.		

## Positron emission tomography tracers for glioma

WHO classification uses molecular parameters in addition to histology for glioma classification, which indicates that non-invasive molecular imaging technic focusing on depicting metabolic processes, such as PET, may be more widely used. In clinical practice, conventional MRI is mostly used for evaluation of gliomas. However, MRI imaging is short of pathology-specific biomarkers and limited by overall diagnostic and prognostic efficacy^[55]^. In the contrary, as a functional molecular imaging, PET can provide additional information about metabolism processes, which are useful for primary and differential diagnosis, delineation of glioma extent, grading, treatment planning, assessment of treatment response and prognosis in gliomas. Currently, more than 70 different PET tracers have been used in brain tumor examinations^[56]^. In this section, we enumerate some representative tracers with the potential of clinical practice.

### ^18^F-FDG

As a glucose analogue, ^18^F-fluorodeoxyglucose (^18^F-FDG) is the most commonly used radiotracer and metabolizes in cells similar to glucose, which makes it possible to use ^18^F-FDG to reflect the glucose metabolism. Abnormal proliferation of cancer cells leads to increased glycolysis, resulting in massive accumulation of ^18^F-FDG. However, the poor tumor-to-background contrast due to the high level of glucose metabolism in normal brain tissue hampers the use of ^18^F-FDG in brain tumors. The differential diagnosis between glioma and other brain tumor, such as CNS lymphoma, is also limited because of their similar metabolic profiles^[57]^ and overlap of maximum standard uptake values (SUV_max_)^[58]^. It's also difficult for ^18^F-FDG-PET to distinguish between glioma and some non-neoplastic diseases such as stroke, MS, and infectious diseases, which are also associated with hypermetabolism^[59]^.

### Radiolabelled amino acids

The malignant proliferation of glioma increases the demand for amino acids and accelerates the rate of metabolism^[60]^. Their increased uptake is caused by the increased transport via the L-type amino acid transporter (LAT) system, especially its subtype LAT1^[61]^. Different than the high distribution of ^18^F-FDG in normal brain tissues, the uptake of amino acid is relatively low, so that gliomas can be distinguished from the low background noise. ^11^C-methyl-L-methionine (^11^C-MET), O-(2-^18^F-fluoroethyl)-L-tyrosine (^18^F-FET) and 3,4-dihydroxy-6-^18^F-fluoro-L-phenylalanine (^18^F-DOPA) are widely used radiolabeled amino acid. They are similar and have the ability of passing through intact BBB, the capacity to differentiate tumor recurrence and treatment effects such as pseudo progression and radiation necrosis after treatment.

#### ^11^C-MET

^11^C-MET is one of the most popular amino acid tracers in glioma PET imaging for its convenient production^[62–63]^. The uptake of ^11^C–MET in cells is mediated by the neutral L-amino acid transporter which serves the increased amino acids utilization of tumor^[64]^. However, the short half-time of ^11^C (20.38 minutes) limits its use only in centers with an on-site cyclotron. Glaudemans *et al*^[65]^ reviewed the value of ^11^C-MET-PET in imaging gliomas. ^11^C-MET has high sensitivity (76%–100%) and high specificity (75%–100%) in diagnosis. It can also be used in tumor delineation with improved diagnose accordance rate, compared to CT and MRI. In biopsy and radiotherapy planning, ^11^C-MET can provide additional information about tumor localization and target volume. A Meta-analysis^[66]^ shows that ^11^C-MET performs well in differentiation of tumor progression after treatment.

#### ^18^F-FET

^18^F-FET and ^11^C-MET provide comparable diagnostic information concerning tumor delineation, biopsy determination and recurrence detection^[67]^. With a long physical half-life (109.77m), ^18^F-FET can be popularized to more hospitals. Yang *et al*^[68]^ show that ^18^F-FET has an average sensitivity of 92% and specificity of 81% in diagnosing glioma. In dynamic PET, time-activity curves of ^18^F-FET are different between low-grade glioma and high-grade glioma^[69–70]^ and provide more additional information for glioma gradingthan ^11^C-MET-PET and ^18^F-FDOPA^[71–72]^.

#### ^18^F-FDOPA

Xiao *et al*^[73]^ demonstrated a pooled sensitivity of 0.90 and specificity of 0.75 using ^18^F-FDOPA for diagnosing gliomas, and a pooled sensitivity of 0.88 and specificity of 0.73 in grading gliomas. However, the small sample size results in inconclusive opinion on its clinical effectiveness for glioma imaging. As an amino-acid PET imaging method, numerous evidence supports its superiority of detecting tumor components, which can be used in tumor recurrence and therapy monitoring, compared to enhanced MRI. However, with contradictory conclusions^[74–76]^, the feasibility for ^18^F-FDOPA to distinguish low-grade glioma and high-grade glioma is yet to be studied. ^18^F-FDOPA is a precursor of dopamine, which may lead to a high uptake in striatum^[61]^.

### 3'-deoxy-3'-^18^F-fluorothymidine

3'-deoxy-3'-^18^F-fluorothymidine (^18^F-FLT) is a thymidine analog, which can be retained in proliferating tissues by the action of thymidine kinase 1 and presents high-contrast images of tumors^[77]^. Early study showed that ^18^F-FLT can detect a larger tumor volume than gadolinium-enhanced MRI imaging in detecting and characterizing brain tumors and provide complementary information together with ^11^C-MET-PET and MRI. ^18^F-FLT can also be used for differentiating recurrent glioma and treatment effects^[78–80]^ but has a limited role by offering no advantages over ^18^F-FDG-PET. ^18^F-FLT can't pass the intact BBB, which limits its usage in tumor delineation and detecting additional metabolic active tumor tissue beyond MRI^[81–82]^.

### Choline

Choline is an essential ingredient for all cells to synthesize biological membrane molecules while abnormal choline aggregation in tumor tissues is observed due to the high proliferation of tumors. There are two kinds of radiolabeled choline tracers to monitor membrane phospholipids: ^11^C-choline (^11^C-CHO) and ^18^F-fluorocholine (^18^F-FHO). Studies with ^18^F-FHO have focused on the evaluation of prostate cancer^[83]^. The low uptake of ^18^F-FHO in normal brain tissues presents a better tumor/normal uptake ratio than FDG-PET^[84]^. Many studies showed that ^11^C- and ^18^F- labeled choline has encouraged results in distinguishing different kinds of brain tumors from metastases and benign lesions^[83]^, evaluating the potential malignancy of glioma^[85]^, guiding stereotactic biopsy sampling^[86]^ and differentiating glioma recurrence^[87–88]^.

### Other tracers

#### ^18^F-fluoromisonidazole

Tumor hypoxia is an interesting mechanism in tumor progress, which may cause the resistant to radiotherapy^[89]^. ^18^F-fluoromisonidazole (^18^F-FMISO) is a PET tracer that selectively binds to hypoxic tissues and can be used to measure the hypoxia in glioma and provide information for prognosis^[90]^. As it is retained by irreversible binding to the thiol-rich metabolic proteins at rates that are inversely proportional to oxygen concentration, ^18^F-FMISO provides quantitative measurements of hypoxia level^[91]^.

#### ^18^F-fluciclovine

^18^F-fluciclovine ( also called anti-^18^F-FACBC) is a synthetic amino acid that has been approved by Food and Drug Administration (FDA) and European Medicines Agency (EMA) for recurrent prostate cancer. Different with other amino acid PET tracers, such as ^11^C-MET, ^18^F-FET and ^18^F-FDOPA, ^18^F-fluciclovine can be transported by both LAT1 and alanine-serine-cysteine (ASC), specifically ASCT2^[92]^. These transporters work as an exchange system that brings in a preferential amino acid at the expense of a less essential one, but do not increase the overall cellular levels of amino acids^[93]^. Many studies have showed that ^18^F-fluciclovine can delineate glioma in areas not visualized using MRI and be used in glioma grading^[92,94–96]^.

#### Acetate and analog

In highly metabolizing and rapidly growing brain tumors, the lack of abundant glucose and oxygen may lead to metabolism reprogramming to obtain sufficient energy, for example, the use of fatty acid^[97]^. Induced acetate/acetyl CoA metabolism is highly related to fatty acid synthesis supporting tumor growth^[98]^. Therefore, many radiolabeled acetates may be used in glioma imaging, such as 1-^11^C-acetate^[99–100]^ (^11^C-ACE), 2-^14^C-acetate^[101]^ (^14^C-ACE), 2-^18^F-fluoroacetate^[102]^ (^18^F-FAC) and ^18^F-fluoropivalic acid^[103]^ (^18^F-FPIA), a newly designed novel radiotracer based on trimethylacetate.

#### ^18^F-FPP(RGD)_2_

Angiogenesis is the process of forming new blood vessels and closely related to tumor growth and progression. The α_v_β_3_ integrin is highly expressed only in activated endothelial cells of tumor neovasculature and can be traced by radiolabeled arginine-glycine-aspartic acid (RGD) peptides and analogs, such as monomeric cyclic ^18^F-galacto-RGD and ^18^F-AH111585. With increased receptor-binding affinity and tumor uptake, 2-fluoropropionyl-labeled PEGylated dimeric RGD peptide (^18^F-FPP(RGD)_2_) is the one used in glioma^[104]^. ^18^F-FPP(RGD)_2_ has increased uptake in glioblastoma multiforme and would be suitable for clinical examinations^[105–106]^.

### Summary

^18^F-FDG is the most widely used PET tracer in oncology but the high uptake in normal brain tissue hampers its clinical use. According to the Response Assessment in Neuro-Oncology group, amino-acid PET is recommended for PET imaging of glioma^[107]^. It does not seem to be one particular tracer that could be recommended over other tracers in all aspects of imaging glioma, maybe even not in any specific usage ^18^. More studies with larger cohort and unified standard are necessary to find the best tracer among all the tracers that have already been used in clinical trials for many years. Some newly designed tracers such as ^68^Ga-Alb-FAPtp-01^[108]^ and ^89^Zr/^177^Lu-labeled Lumi804-aCD11b can be used both in imaging and treatment^[109]^. Tracers for glioma imaging are summarized in ***Table 2***^[62–63,65–90,92,94–96,99–106]^.

**Table 2:** A summary for positron emission tomography tracers used for glioma

Chemical type	Tracer	Target or intake mechanism	Note
Glucose analogue	^18^F-FDG	Glucose	Demonstrate glucose metabolism; Poor tumor-to-background contrast
Amino-acid	^11^C-MET^[62–63,65–66]^	LAT1	Demonstrate amino-acid uptake; High sensitivity and specificity; Best and most studied tracers for glioma
	^18^F-FET^[67–72]^		
	^18^F-FDOPA^[73–76]^		
Thymidine analog	^18^F- FLT^[77–82]^	Thymidine kinase 1	Markers of cell proliferation; Cannot pass intact BBB
Choline	^11^C-CHO^[84–86,88]^	Choline transporter	Monitor membrane phospholipids; Cannot pass intact BBB
	^18^F-FHO^[83,86–87]^		
Nitroimidazole	^18^F-FMISO^[89–90]^	Nitroreductase enzymes	Investigate intratumoural hypoxia; Haven't achieved clinical relevance
Amino acid	^18^F-fluciclovine ^[92,94–96]^	LAT1 & ASCT2	Demonstrate amino-acid uptake; Transported by both LAT1 and ASCT2
Acetate	^11^C-ACE^[99–100]^	Acetate/acetyl CoA metabolism	Demonstrate the use of fatty acid
	^14^C-ACE^[101]^		
	^18^F-FAC^[102]^		
	^18^F-FPIA^[103]^		
Peptide	^18^F-FPP(RGD)_2_^[104–106]^	αvβ3-integrin	Demonstrate angiogenesis
^18^F: ^18^fluorine; ^11^C: ^11^carbon; FDG: fluorodeoxyglucose; LAT1: L-type amino acid transporter-1; MET: methyl-L-methionine; FET: O-(2-^18^F-fluoroethyl)-L-tyrosine; FDOPA: 3,4-dihydroxy-6-^18^F-fluoro-L-phenylalanine; FLT: 3′-deoxy-3′-^18^F-fluorothymidine; CHO: choline; FHO: fluorocholine; FMISO: fluoromisonidazole; ASCT2: alanine-serine-cysteine-2; ACE: acetate; FAC: fluoroacetate; FPIA: fluoropivalic acid; FPP(RGD)_2_: 2-fluoropropionyl-labeled PEGylated dimeric RGD peptide; BBB: blood-brain-barrier.			

## Positron emission tomography tracers for Alzheimer's disease

AD is the most common type of dementia. It makes up about two-third of all differential diagnoses about dementia. About 10% of people elder than 65 are thought to have AD^[110–111]^. As a progressive and irreversible neurodegenerative disorder, patients with AD suffer from gradual loss of memory and progressive cognitive dysfunction involving the language, visuospatial and executive domains. In 1970s, cholinergic hypothesis of AD was proposed according to the essential role of acetylcholine in cognitive function. However, the most accepted hypothesis of AD is amyloid cascade hypothesis^[112]^, which posits that amyloid-β (Aβ) accumulation is the primary event leading to a cascade of effects that result in neuronal damage while hyperphosphorylated protein (*e.g.*, p-Tau) tangles, the second hallmark of AD, is a downstream effect of Aβ accumulation. Therefore developing associated agents for detecting these AD abnormal hallmarks in the brain may be helpful for the early and differential diagnosis of the disease progression and prognosis evaluation.

### Amyloid-β plaques

The golden-standard diagnosis of AD is based on autopsy while 40% patients may be misdiagnosed under current *in vivo* clinical diagnosis criteria because of the difficulties in accurate discrimination of AD and non-AD dementia^[113]^. According to the National Institute on Aging and the Alzheimer's Association, AD could be defined and studied in terms of three stages: preclinical, mild cognitive impairment (MCI) and dementia^[114]^. It is suggested to prevent AD in the preclinical phase, rather than trying to cure it when clinical symptoms have appeared.

In AD patient, the balance between production and clearance of Aβ is broken, which causes the abnormal accumulation and result in aggregates with β-pleated sheet structure^[115]^. Extracellular Aβ oligomers bind the cell surface, leading to receptors' functional of disruption and finally cause the synaptic dysfunction and neurodegeneration^[116]^. The Aβ accumulation comes far before the clinical symptoms and may be used for early prediction of AD. The level of Aβ in cerebrospinal fluid (CSF) has been recommended as a usable and cost-less tool in the diagnosis and monitoring of AD by the European Medicines Agency^[117–118]^. PET imaging with Aβ targeted radiotracers can hopefully identify potential AD patients and monitor its progression in a non-invasive and safer way.

#### The first generation

^**11**^**C-PIB:** The earliest and most widely used and studied radiotracer for amyloid PET imaging is N-methyl-^11^C-2-(4′-methylaminophenyl)-6-hydroxybenzothiazole, a benzothiazole derivative, also called ^11^C-Pittsburgh compound B (^11^C-PIB)^[119]^. It is derived from Thioflavin T (Th-T), a histopathological dye that binds to the amyloid plaque, as well as Congo Red. ^11^C-PIB can easily cross the BBB and bind to amyloid plaques with high affinity. It is proved that ^11^C-PIB retention in the frontal cortex and posterior cingulum significantly correlate with CSF Aβ^[120]^, and can be used to predict the disease progression from MCI to AD^[121–123]^.

^**18**^**F-flutemetamol:** ^18^F-flutemetamol (Vizamyl, also known as ^18^F-GE067 and 3'-^18^F-PIB) is developed in the seeking of ^18^F labeled radiotracer similar to ^11^C-PIB but can be popularize to centers without cyclotron. Many studies have shown that the uptake processes and specific binding characteristics of flutemetamol is very similar to ^11^C-PIB^[124–125]^. It has been approved by FDA in 2013.

^**18**^**F-florbetapir and**^** 18**^**F-florbetaben**: ^18^F-florbetapir (Amyvid, also known as ^18^F-AV45 or florpiramine) and ^18^F-florbetaben (Neuraceq, also known as ^18^F-AV-1 or ^18^F-BAY94-9172) are both diaryl alkene analogs. In this branch, ^3^H-SB-13 is the first stilbene derivative with high affinity to Aβ^[126]^, and then led to its ^11^C-labeled analog ^11^C-SB-13^[127]^. ^18^F-florbetapir is the first ^18^F-labeled PET tracer developed for imaging amyloid plaques and has been approved by FDA in 2012. ^18^F-florbetaben was approved by FDA in 2014.

The three FDA approved amyloid tracers^[113]^ of ^18^F-flutemetamol, ^18^F-florbetapir and ^18^F-florbetaben all show high correlation with ^11^C-PIB and can be used in the prediction of MCI progression and diagnosis of AD. Since nearly all the studies about those tracers are compared with ^11^C-PIB, it is hard to deny the hypothesis that they are quite similar in sensitivity and specificity. The biggest problem of the first generation of Aβ pet tracers is the low signal-to-background ratio, which limits the sensitivity especially in the early phase AD patients with low plaque levels. Hence, many second-generation tracers were developed to solve this problem.

#### The second generation

^**18**^**F-flutafuranol:** ^18^F-flutafuranol (also known as ^18^F-AZD4694 or ^18^F-NAV4694) is a benzofuran derivative firstly synthesized in 2010^[126]^. Same as the development of ^18^F-florbetapir and^ 18^F-florbetaben, the first tracer in this branch was also ^3^H labeled, ^3^H-AZD2184, which displays a twice higher signal-to-background ratio than ^3^H-PIB^[128]^. The high signal-to-background ratio is also observed on ^11^C-AZD2184^[129]^. ^18^F-flutafuranol shares the identical binding with the second-generation tracers and shows a lower non-specific binding *in vitro*. Following studies show that ^18^F-flutafuranol has a high cortical binding in AD, highly correlating to ^11^C-PIB, and lower non-specific white matter binding, which confirmed its potential for wide clinical application^[130–131]^.

### Tau aggregates

According to the amyloid cascade hypothesis, the generation of Aβ plaques is the primary progression of AD and causes other downstream events, such as Tau aggregates. However, amyloid volume has no additional value on disease severity^[132]^. On the contrary, the extent of neurofibrillary tangles composed of Tau, seems to be increased with disease progression and is closely related to memory decline^[133]^. Therefore, Tau PET is used for both early diagnosis and tracing disease progression.

^**18**^**F-FDDNP:** ^18^F-FDDNP is the first PET tracer labelling regional Tau tangles and shows the ability of differentiating MCI from normal aging and AD^[134]^. However, its relatively high affinity to Aβ limits the Tau imaging specifically^[135]^. In order to improve the specificity and selectivity, many other sets of tracers were synthetized and referred to as the first generation.

#### The first generation

**Quinoline derivatives [2-(4-aminophenyl)-6-(2-fluoroethoxy)quinoline] [THK] series):** Based on the new quinoline and benzimidazole derivatives that bind Tau over Aβ plaques found by Okamura *et al*^[136]^, the first tracer of the THK series of ^18^F-THK523 was developed and fulfils ligand criteria for human imaging trials^[137]^. Afterwards, ^18^F-THK5105 and ^18^F-THK5117 are found to have a higher binding affinity than ^18^F-THK523^[138]^ but have a relative high retention within the white matter^[139]^. The latest tracer in this series is ^18^F-THK-5351 (also known as GE-216) with faster kinetics, higher contrast, and lower retention in subcortical white matter than^18^F-THK5117^[140]^. Many studies have shown that the THK series have a high off-target binging to MAO-B, which accumulates with age, and hence, may be limited in the use of Tau specific imaging.

**The carbazole and benzimidazole derivatives (**^**18**^**F-flortaucipir):** ^18^F-flortaucipir (also known as Tauvid, ^18^F-AV1451 or ^18^F-T807) is a benzimidazole pyrimidine derivatives developed in 2013 and has high affinity, selectivity, and lipophilicity^[141]^. It provides high contrast because of low retention in white matter. Clinical studies show that ^18^F-flortaucipir PET present significant correlations with CSF measurements of tau pathology^[142]^ and is associated with clinical impairment^[143]^. ^18^F-flortaucipir has been approved by FDA for Tau imaging in 2020. However, ^18^F-flortaucipir also has similar affinity to MAO-B, like the THK-series^[144]^.

^**11**^**C-PBB3:** ^11^C-PBB3 (pyridinyl-butandienyl-benzothiazole 3) is another tracer for Tau^[145]^. ^11^C-PBB3 PET has a robust signal in Tau enriched tissue and is consistent with the spreading of Tau pathology with AD progression. It also has a low white matter binding similar as ^18^F-flortaucipi while the radiometabolites may remain in the brain and hamper its quantification.

#### The second generation

There are some common limits in the first generation of Tau tracers, such as retention in white matter and off-target binding especially to MAO-B. Therefore, the second generation now enters the field. Some of them are improved from reported tracers and have a similar structure as the first generation.

**Subsequent ^18^F-flortaucipir series:** Based on ^18^F-T808 (also called ^18^F-AV680), which is in the same series with ^18^F-flortaucipir but abandoned for its propensity to metabolic defluorination^[146]^, ^18^F-GTP1 (^18^F-Genentech tau probe 1) was developed without measurable binding to MAO-B and evidence of defluorination^[147]^. Based on ^18^F-flortaucipir, three compounds were developed with lower affinity to MAO-B than ^18^F-flortaucipir and THK-series, in which ^18^F-RO948 (^18^F-RO6958948) was found to have a better pharmacokinetics and metabolic properties and higher signal-to-background ratio^[148]^. ^18^F-PI-2620 is another Tau tracer improved from ^18^F-flortaucipir without off-target binding to MAO-B^[149]^.

**^18^F-PM-PBB3:** Based on ^11^C-PBB3, ^18^F-PM-PBB3 (^18^F-APN-1607) has developed with higher metabolic stability and less off-target signals, which makes it higher signal-to-background ratio^[150]^. ^18^F-PM-PBB3 also correlated well with cognitive changes^[151]^.

^**18**^**F-MK6240:** ^18^F-MK6240 is an azaindole derivatives developed in 2016^[152]^. It can efficiently differentiate normal control group, MCI and AD patients. No defluorination or off-target signals to MAO-B has been observed^[152]^. Clinical trials also showed a minimal off-target binding in human brain^[153]^.

^**18**^**F-JNJ311:** ^18^F-JNJ311 (^18^F-JNJ64349311) is a naphthyridine derivatives developed in 2017^[154]^. Animal studies showed its moderate initial brain uptake, rapid brain washout, and minor off-target specific binding in the healthy rat brain^[155]^. ^18^F-JNJ311 has a favorable pharmacokinetic profile like ^18^F-flortaucipir and is a potential candidate for Tau-specific tracer.

Tracers for Alzheimer's disease are summarized in ***Table 3***^[113,119–126,128–131,134–135,137–142,144–145,147–155]^.

**Table 3:** A summary for positron emission tomography tracers used for Alzheimer's disease

Target	Generation	Tracers	Note
Aβ			
Planar heteroaromatic analogs	1	^11^C-PIB^[119–123]^	Classical used tracer
	1	^18^F-flutemetamol^[124–125]^	FDA approved
	1	^18^F-florbetapir^[113]^	FDA approved
	1	^18^F-florbetaben^[113]^	FDA approved
	2	^18^F-flutafuranol^[126,128–131]^	
Tau			
Naphthalene analogs	0	^18^F-FDDNP^[134–135]^	Affinity for both Aβ and Tau
Arylquinoline derivatives (THK series)	1	^18^F-THK-523^[137]^	
	1	^18^F-THK-5105^[138]^	
	1	^18^F-THK-5117^[138]^	
	1	^18^F-THK-5351^[140]^	
Benzimidazole pyrimidines derivatives	1	^18^F-flortaucipir^[141–142,144]^	FDA approved
	2	^18^F-RO948^[148]^	
	2	^18^F-PI2620^[149]^	
	2	^18^F-GTP1^[147]^	
PBB	1	^11^C-PBB3^[145]^	
	2	^18^F-PM-PBB3^[150–151]^	
Azaindole derivatives	2	^18^F-MK6240^[152–153]^	
Naphthyridine derivatives	2	^18^F-JNJ311^[154–155]^	
Aβ: amyloid-β protein; ^18^F: ^18^fluorine; ^11^C: ^11^carbon; PIB: Pittsburgh compound B; FDDNP: 2-(1-{6-[(2-fluoroethyl)(methyl)amino]-2-naphthyl}ethylidene)malononitrile; PBB3: pyridinyl-butadienyl-benzothiazole 3; FDA: Food and Drug Administration.			

## Conclusions

There are many hurdles in developing PET tracers for glial cells and related disease. For example, a candidate tracer must have the ability to across BBB. After entering the CNS, it must have sufficient affinity and specificity to its target with low off-target binding. In the field of glial tracer imaging, future work should focus on the different phenotypes of microglia and astrocytes. It's clear that there is more complex and precision differentiation, which can be a great progress to understand the precise roles of glial cells in neuroinflammation and many other related diseases. Some advanced technology can be helpful for development of new glial tracers, such as computational BBB permeability modeling tools, human BBB microfluidic and BBB organoids.

Furthermore, new imaging systems may bring a revelation of glial imaging with better resolution and sensitivity. PET/MRI is a technology that combines the anatomic and quantitative strengths of MRI with physiologic information obtained from PET. Compared with PET/CT, it has an increased soft tissue contrast and lacks of ionizing radiation exposure, which outperforms PET/CT in many applications^[156]^. It has been used in both neurodegenerative disorders^[157–159]^ and glioma^[160–162]^, with above-mentioned radiotracers, PET/MRI has achieved much better results than using PET or MRI individually, and also PET/CT.
